# Learning a Transform Base for the Multi- to Hyperspectral Sensor Network with K-SVD

**DOI:** 10.3390/s21217296

**Published:** 2021-11-02

**Authors:** Thomas Hänel, Thomas Jarmer, Nils Aschenbruck

**Affiliations:** Institute of Computer Science, Osnabrück University, 49090 Osnabrück, Germany; tjarmer@uos.de (T.J.); aschenbruck@uos.de (N.A.)

**Keywords:** compressed sensing, multispectral imaging, precision agriculture, wireless sensor networks

## Abstract

A promising low-cost solution for monitoring spectral information, e.g., on agricultural fields, is that of wireless sensor networks. In contrast to remote sensing, these can achieve more continuous monitoring due to their long-term deployment and are less impacted by the atmosphere, making them a promising solution for the calibration of satellite data. In this paper, we explore an alternative approach for processing data from such a network. Hyperspectral sensors were found to be too complex for such a network. While previous work considered fusing the data from different multispectral sensors in order to derive hyperspectral data, we shift the assessment of the hyperspectral modeling in a separate preprocessing step based on machine learning. We then use the learned data as additional input while using identical multispectral sensors, further reducing the complexity of the sensors. Despite requiring careful parametrization, the approach delivers hyperspectral data of similar and in some cases even better quality.

## 1. Introduction

The continuous monitoring of plants at high spatial and temporal resolution is a crucial component of making agriculture more efficient and thereby preparing it for an increasing world population. Currently, such information is mainly collected by remote sensing or when agricultural machines drive on the field. However, while offering high spatial resolution, these methods are not suitable for continuous monitoring at high temporal resolution.

An element for continuous monitoring could be added in the form of a Wireless Sensor Network (WSN) with nodes that include multiple sensors. One very versatile sensor for such a node would be an optical spectrometer or hyperspectral sensor. The versatility stems from the fact that a multitude of information on the plant condition can be derived from different ranges of the spectrum. Naturally, more spectral bands allow for the extraction of more information. We argue that obtaining spectral data may even be superior to adding many specialized sensors as this reduces the cost of individual sensor nodes, allows for re-using plenty of models built around remote sensing data, and allows for the calibration of remote sensing data that contain similar information.

In previous works [1,2], it was determined that multispectral sensors with less than 10 bands are more likely to be affordable for such a network than actual spectrometers with significantly more bands. While the miniaturization and cost reduction of such spectrometers are also marking significant steps forward, we expect that multispectral sensors will always stay ahead due to their lower complexity. Smart dust is an idea for the more distant future. In agriculture, it encompasses large numbers of sensor nodes being "planted" together with the plants. If development actually moves in that direction, we expect multispectral sensors may reach a sufficiently small size sooner than hyperspectral sensors and many more specialized sensors. Due to the huge number of devices, reducing the complexity and cost of the individual sensor nodes becomes even more important in that case.

When deriving vegetation information, naturally, high-resolution spectra as acquired by hyperspectral sensors allow for the calculation of more information than multispectral sensors with lower spectral resolution. When using multispectral sensors, there are multiple ways to overcome these limitations: firstly, one may simply accept having less information available; secondly, one may research new algorithms and metrics based on the limited number of bands, yielding similar information. However, this needs to be repeated for every new set of bands; thirdly, one may add a step in between consisting of calculating a high-resolution spectrum based on the low-resolution spectrum and deriving information from it. This allows for re-using all the algorithms and metrics designed for hyperspectral data, making the sensors on the one hand and the algorithms and metrics on the other hand more replaceable. This third approach also facilitates the calibration of remote sensing data as matching bands can be constructed.

**Figure 1:**
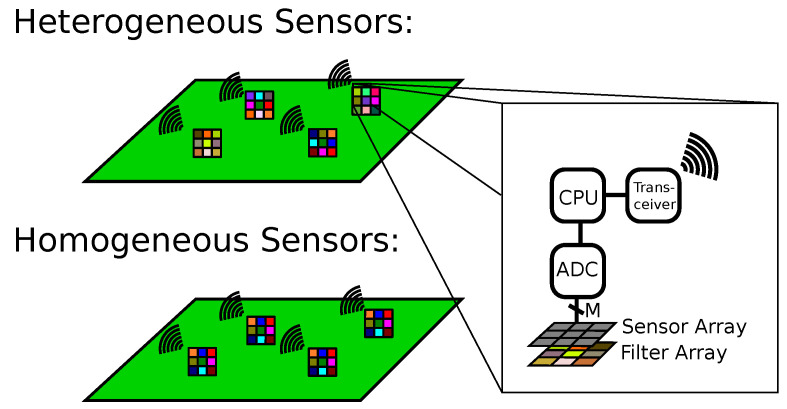
Architecture of an M2HSN with a heterogeneous and homogeneous choice of bands.

In this paper, we further investigated the third way which we called Multi- to Hyperspectral Sensor Network (M2HSN) in [2]. The structure of an M2HSN is shown in Figure 1. It consists of multiple sensor nodes with each node being equipped with an array of light sensors whose readings are digitized and transmitted to a fusion center. These sensor nodes are turned into multispectral sensors by adding different optical filters in front of the sensor array. Thus, the sensor nodes are very simple in comparison to high-resolution spectrometers. Note that this also lowers the amount of data transmitted to the fusion center in comparison to actual high-resolution spectrometers.

There are two possibilities for choosing the filters: these may either be homogeneously chosen with the same set of filters in every node or heterogeneously with varying filter sets at different positions.

In our previous work [2], we focused on the heterogeneous case. In this paper, we investigated the homogeneous case. As no customization of the hardware is required with respect to the location, the mass fabrication of such sensors will be more feasible. These are in fact similar to sensors already being cheaply available nowadays. In contrast to the heterogeneous case, the M2HSN with homogeneous filter sets does not gather any information on the bands not included in the band set. The data must instead be obtained from a different source. One may argue, that this is a problem of the approach, because obtaining hyperspectral data is not fully avoided. Therefore, we evaluated the feasibility of supplying this information by learning it from remote sensing data. The remote sensing data for training needs to be hyperspectral; however, no hyperspectral measurements are required on the ground. As the M2HSN is mainly intended as an addition to remote sensing, the less frequently obtained remote sensing data can be used as training data for the M2HSN. It is therefore relatively easy to obtain. The algorithm for learning the information is K Singular Value Decomposition (K-SVD) [3].

K-SVD was previously used for estimating hyperspectral images from RGB images as acquired by smartphone cameras or DSLRs [4]. However, to the best of our knowledge, it has never been investigated how well this approach applies to remote sensing data and data collected on the ground in agricultural fields. This is one of the main areas investigated using spectral remote sensing which we believe deserves an isolated investigation. Furthermore, we investigated how the approach benefits from adding more bands in a broader range of wavelengths—this seems a logical choice as bands in the near infrared proved essential for vegetation.

Our core contributions in the paper are: (1) the first evaluation of the suitability of increasing spectral resolution with K-SVD for in situ and remote-sensing data; (2) providing the idea of the homogeneous M2HSN and its simulative evaluation; and (3) offering a guide for choosing the correct kind of M2HSN, data-processing algorithm, and parametrization depending on the scenario.

The remaining part of this paper is structured as follows: in Section 2, we give an overview of the underlying methods used and evaluated in this paper and outline the embedding of our work into current research; Section 3 contains a description of how the methods are used and modified; in Section 4, we evaluate the configuration of K-SVD in-depth; the comparative evaluations with all methods follow in Section 5; and finally, we draw conclusions in Section 6.

## 2. Background and Related Work

### 2.1. Gaining Information from Vegetation Spectra

The spectra of plants contain plenty of information on their status. A basic example is assessing how green a plant is, which the human eye is capable of. In the remote sensing community, this was expressed more formally with vegetation indices such as the Visible-Band Vegetation Index (VDVI), Normalized Green-Red Difference Index (NGRDI), and the Normalized Green-Blue Difference Index (NGBDI) [5] which are based on a red, a green and a blue band or a subset thereof. However, the reflection of vegetation is much higher in the near infrared. Therefore, the Normalized Difference Vegetation Index (NDVI) [6], which uses a red band and a near infrared band, became widely used. The steep increase in reflection at approximately 700 nm is known as the red edge [7]. It has been used in the calculation of another vegetation index, the Normalized Difference Red Edge Index (NDRE) [8].

However, the exact choice of the bands used for the calculation of these vegetation indices is not defined as it depends on the sensors, and has a significant impact on vegetation indices, which was shown for the NDVI [9]. Gaining higher resolution spectra allows for choosing the exact bands for the calculation of indices later on. An online database [10] lists more than 200 vegetation indices based on many different bands. While low-resolution spectra only contain small subsets of bands for small subsets of these indices, all indices which lie in the spectral range of a high-resolution spectrum are calculable from it. While the individual indices typically require relatively few bands, for the determination of the inflection point of the red edge, multiple bands at high resolution around the red edge are required [11].

In addition to the expansion to a greater selection of vegetation indices, another application that benefits from high-resolution spectra is the classification of plant species [12]. As sensing actual hyperspectral data is not always possible or affordable, we investigated the estimate of hyperspectral data based on multispectral data in this paper.

The quality of this approach may be evaluated in different ways, e.g., the impact on classification metrics such as recall and precision or the error introduced in the vegetation indices may be compared against those observed with actual hyperspectral data. However, it always makes sense to first directly compare the reconstructed high-resolution spectra against original high-resolution spectra. Therefore, we concentrated on both qualitatively and quantitatively characterizing the quality of the reconstructed spectra. The impact on various classification problems and vegetation metrics is beyond the scope of this paper.

### 2.2. Compressed Sensing

The enabling theory at the core of some of the following methods is compressed sensing [13,14]. It allows for sensing only a fraction of data and later reconstructing the whole data. In other words, it allows for the merging of the established operations of sensing and compression into a single operation, hence the name. In contrast to the established separate operations, the necessity of throwing away data during compression which has been sensed at high cost is eliminated—the reduction already takes place during sensing. Finding a solution becomes possible by incorporating a small amount of expert knowledge in the form of assuming the sparse signal in a given domain which is known to be valid in many applications. In many cases, the modification of the sensing process is as easy as sensing at random times, locations, or in the context of this paper, spectral bands [15].

### 2.3. Distributed Compressive Sensing (DCS)

DCS [16] is an approach for handling multiple similar signals as they are acquired in a WSN and multiple locations. A typical example of such signals in WSNs are sensor values over time. In the context of the M2HSN, it is a spectrum instead. DCS specifies multiple kinds of similarity, called Joint Sparsity Models (JSMs). Thereof, only the first model, called JSM-1, is relevant in the rest of the paper. It foresees a sparse common signal and a sparse innovation signal for each location, which is added to the common signal.

### 2.4. Universal Pattern Decomposition Method (UPDM)

UPDM [17] is an approach that stems from remote sensing. It has been used for simulating data from hyperspectral satellites based on data from multispectral satellites. The core idea of UPDM is modeling the spectrum as a composition of multiple spectra from different materials, namely water, vegetation, and soil. Based on the multispectral data, the fraction of the materials in the mix is estimated. Using the high-resolution spectra of each of the materials and the fractions in each pixel, the hyperspectral data are then calculated.

### 2.5. K Singular Value Decomposition (K-SVD)

In the earlier works on compressed sensing, mostly long-established transform bases such as Discrete Cosine Transform (DCT), Discrete Fourier Transform (DFT) and Discrete Wavelet Transform (DWT) [13] were considered and soon recombined to construct more specialized transforms as with Kronecker Compressive Sensing (KCS) [18] and DCS [16]. K-SVD [3] represents a trend towards even more specialized transforms by learning a very well-fitted transform based on existing data. The learning process for this transform base or dictionary consists of two steps which are repeated until the solution converges. In the first step, a sparse solution for the training data based on the current dictionary was determined using a compressed sensing solver. In the second step, the dictionary is improved by replacing elements of the dictionary using singular value decomposition.

### 2.6. Multi- to Hyperspectral Sensor Network (M2HSN)

The idea of deriving hyperspectral data from a WSN was first proposed in our previous work [2]. The core principle was having a network with multispectral sensor nodes that sense different sets of bands at different places. We argued that it would only require the minor customization of sensor nodes because of just adding different filters. In processing, both the typical spectrum across the whole area and the difference from this spectrum at each position is estimated using the Joint Sparsity Model 1 (JSM-1) of DCS [16].

In comparison to this approach, with K-SVD, the modeling of a typical common spectrum is shifted forward from the data processing step to a separate training step. As the information about the spectra is then already contained in the transform and does not need to be fully derived from the data, the bands can remain fixed, reducing the customization necessary on the nodes even further.

### 2.7. From RGB to Hyperspectral

One other research group attempted to apply K-SVD to calculate hyperspectral data and impressively demonstrated its success on RGB images from DSLR cameras [4,19]. The photos considered were those typically taken with such cameras, e.g., landscape and architecture. Here, we use a very similar approach. However, the kind of data we considered, i.e., ground-measurements and remote sensing data, is very different from those examples. The spectral range is wider, including the near infrared which is of particular interest in vegetation applications and in which the number of bands is not fixed to three. We specifically focused our research on how the approach performs with this kind of data as it is one of the prime application areas for hyperspectral data.

## 3. Materials and Methods

We compared the three approaches—DCS (more specifically, the variant JSM-1), K-SVD, and UPDM—on two datasets: one measured on the ground and one measured in the air. In this section, we provide more details on how we use the methods.

### 3.1. Distributed Compressive Sensing (DCS)

DCS, or more specifically, JSM-1, is mostly used as described in [2], i.e., with a first-degree differential matrix for both the common part as well as the innovation signals which represent the difference between the common part and the individual nodes. Using such a differential matrix means assuming sparsity in the derivative of the spectrum which is a good assumption because the spectra are relatively smooth. The solver in use is Smoothed $\ell_{0}$ (SL0) [20]. In order to limit calculation times, we calculate DCS on groups of 64 spectra. In [2], a possible future improvement was suggested: clustering groups of similar spectra to achieve improved results. This is a non-trivial problem as it would require assessing the quality of the groupings in an iterative approach. However, getting quality estimates in compressive sensing is relatively hard as compressive sensing already squeezes an astonishing amount of information out of small amounts of data. Obtaining a quality estimate would mean squeezing even more information out of the same small amount of data. Therefore, we proposed and evaluated a related approach that is more simplistic and does not require a quality estimate.We performed the grouping *L* times and the DCS calculation for each of these groupings, obtaining *L* spectra per pixel. We then calculated the median of the *L* band value estimates for each band, resulting in a median spectrum. This helps discarding the relatively few bad estimates of the spectra. Note that this is only applicable in a large-scale sensor network.

### 3.2. K Singular Value Decomposition (K-SVD)

K-SVD is used similarly to the approach in [4]: a high-resolution dataset is used to train a transform base for compressed sensing. The resulting transform matrix consists of some artificial spectra that are well suited for sparse representations of the spectra in the training set. We used a Python implementation of K-SVD, https://github.com/nel215/ksvd, (accessed on 27 October 2021) that internally used Orthogonal Matching Pursuit (OMP) [21] as a solver. The resulting transform matrix was then used for the recovery of the high-resolution spectrum in an ordinary compressed sensing approach. In this step, we used SL0 [20]. As K-SVD, in contrast to DCS, does not require heterogeneous band selection, we used a simple selection of best bands. In contrast to [19], we did not use a genetic algorithm for two reasons: firstly, a genetic algorithm would introduce many adjustable parameters, making the solution excessively dependent on finding good parameter sets for each dataset. For our scenario, we considered this an unjustified overhead considering the small increase in performance found in [19]. Secondly, we will show that it is more important to discard bad band sets than trying to find a near-optimal selection—this is also in line with the original idea of making random samples in compressed sensing. Discarding these is much easier: we simply try a few random band sets on a different dataset and select one of the good ones.

### 3.3. Universal Pattern Decomposition Method (UPDM)

UPDM is used as specified in [2], i.e., the base spectra for water, vegetation, and soil are selected from the map in their hyperspectral form. The multispectral form is directly derived by selecting the corresponding bands. Then, at each pixel from the low-resolution spectrum, the composition of water, vegetation and soil is calculated. The high-resolution base spectra are then combined in the same way. Instead of using different band sets at different pixels, we use the same approach for selecting a good band set as for K Singular Value Decomposition (K-SVD).

### 3.4. Datasets

In this work, we considered two of the datasets considered in [2]: one with the spectra acquired with hand-held sensors on the ground, containing only spectra from agricultural fields; and the second dataset whose data was captured by airplane, containing an area mostly consisting of agricultural fields. For the following evaluations, we worked with these sub-datasets:

#### Air_Full

This is the complete air-based dataset.

#### Air_MA, Air_MB

These are two sub-datasets acquired by cutting *Air_Full* into two parts of equal size. The *M* stands for *Mixed* as they contain different kinds of land-use.

#### Air_M1, Air_M2, Air_M3

These are three sub-datasets acquired by cutting *Air_Full* in three parts of equal size. The *M* stands for *Mixed* as they contain different kinds of land use.

#### Air_V1, Air_V2

These are two sub-datasets acquired by cutting squares out of *Air_Full*, which solely contain vegetation.

#### Ground_Full

This is the complete ground-based dataset.

#### Ground_V1, Ground_V2

These are obtained by cutting *Ground_Full* into two parts of equal size.

These datasets will be used as training datasets, as datasets for the selection of best bands and for the evaluation of the algorithms. Note that the air-based dataset and the ground-based dataset originally contained different bands. In order to make them inter-operable, we limited the range of wavelengths to those included in both sensors, from 406 nm to 1100 nm. We then re-sampled the ground-based dataset selecting the same bands as in the air-based dataset. This direction minimizes the re-sampling error due to the higher spectral resolution of the ground-based dataset.

## 4. Parametrization

**Figure 2:**
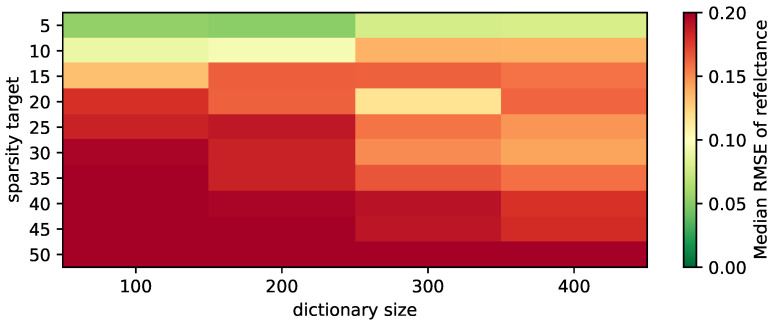
Impact of different dictionary sizes and sparsity targets on reconstruction quality.

In this section, we focused on the evaluation of K-SVD because it is the method which is newly introduced to this kind of data in this paper. The goal here was to develop an understanding of how K-SVD manages to cope with the data and find an appropriate parametrization. In a first step, we determined the size of the dictionary and the appropriate value for the sparsity target. We used a similar value range to the plot in [4] in order to allow for a comparison. The result is shown in Figure 2. The sparsity target was varied from 5 to 50 and the dictionary size from 100 to 400. The training was performed on *Air_M1*, the best bands were selected using *Air_M2* and the evaluation was performed on *Air_M3*. The color shows the median Root Mean Square Error (RMSE) which we determined by first calculating the RMSE for the spectrum of each pixel in the evaluation area. We then determined the median across all pixels. Finally, we repeated the process 20 times with different seeds for the band selection and obtained the median of the 20 medians. Note, that we deferred a closer investigation of the distribution of the RMSEs to Section 5.

Curiously, the result drastically differs from that in [4]: the error increases when increasing the sparsity target. Increasing the dictionary size only increases reconstruction for higher sparsity targets. However, as the previous effect is stronger, best results are therefore achieved with small dictionary and low sparsity target. We attribute this to the spectra varying less across pixels because of the comparably low resolution in remote sensing, which leads to averaging out special spectra, and because of the relatively homogeneous agricultural environment.

**Figure 3:**
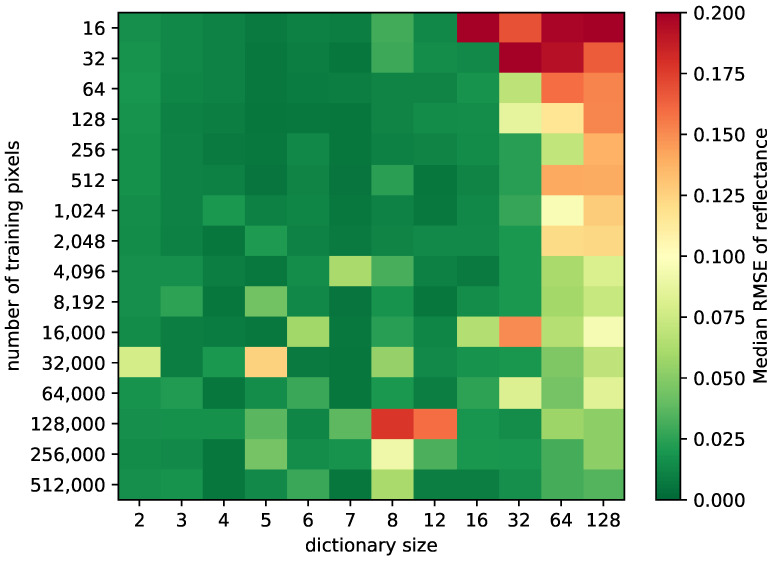
Impact of different dictionary sizes and sizes of the training data on reconstruction quality.

Having found that a very small dictionary suffices, such a small dictionary should be trainable from a smaller training dataset. Therefore, we evaluated the training dataset size in the following step by simply selecting a limited number of pixels at random from the training dataset. This is shown again in Figure 3 which displays the median RMSE of the reflectance. Instead of the sparsity target, we now vary the number of training pixels. The sparsity target is set to 10 percent of the number of bands in the hyperspectral version of the spectrum. The 10 percent rule is the default rule of the K-SVD implementation in use. The resulting sparsity target is 4, which is close to the optimum in Figure 2. Furthermore, the sparsity target is limited to the dictionary size as it is impossible to choose more atoms than there available. From Figure 3 it becomes clear that, surprisingly, a small number of training pixels suffice for a reflectance RMSE of less than approximately 0.03. Increasing the number of training pixels mainly helps increase the result with larger dictionaries. However, as already seen in Figure 2, a large dictionary leads to a lower reconstruction quality. For dictionary sizes below approximately 16, the results are quite good. Dictionary sizes of 2 and 8 are slightly worse. At the value of 2, we attribute this to the dictionary simply being too small. At the value of 8, we found out that this happens due to SL0 performing poorly when the dictionary size is equal the number of bands. The effect does not occur with OMP—but we still stick with SL0 because of an overall better reconstruction quality. Curiously, the reconstruction quality becomes more variable with the increasing training set size. We attribute this to an increasing chance of having anomaly pixels in the training set. Just a few of these suffice to create a transformation that tries to cover the anomaly pixels as well.For smaller training sets, this can happen in rare cases and will have an even worse effect.However, these cases are not reflected in this evaluation plot as they are rejected when the median is calculated.

**Figure 4:**
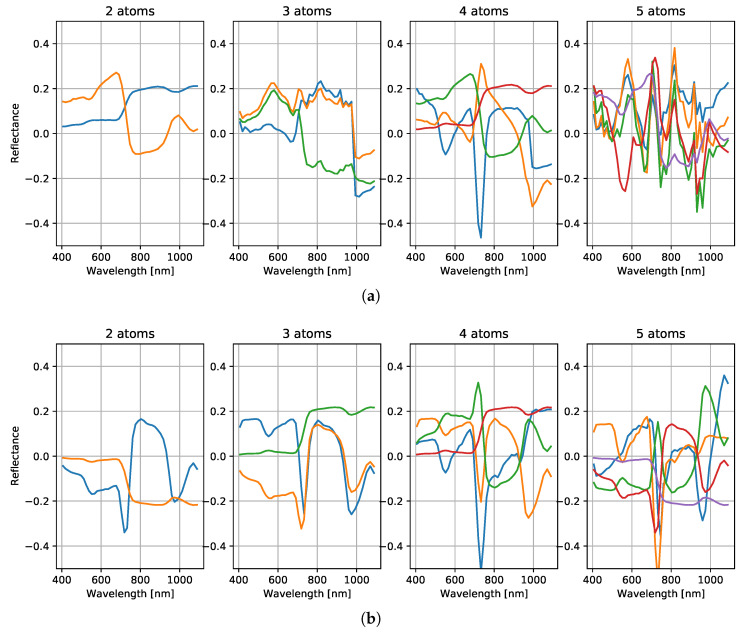
Atoms trained at different dictionary sizes and on different training sets. (**a**) Trained on Air_Full. (**b**) Trained on Ground_Full.

An advantage of such a small dictionary is that it can be visualized for qualitative investigation. Some samples are shown in Figure 4. Each plot contains the elements of a trained transform, also called atoms, as line plots. The number of atoms is increased from left to right by re-training with a different dictionary size. The atoms have different colors merely for visualization, and the order is arbitrary. Clearly, most atoms are dominated by the red edge and adding more atoms mainly helps refine the representation of the red edge. A comparison of Figure 4a,b, shows that this effect may be observed for both datasets. Note that the dimension of this basis is comparable to the one assumed in UPDM with its three-base spectra. Due to small number of atoms, which are usually all present in the solution, even using an $\ell_{2}$-solver becomes viable, turning the approach into a more simplistic approach. However, we still use SL0 which starts with the $\ell_{2}$-solution anyway and can thereby be considered a more general solver. Note that this model of the spectra including a linear combination of a few base spectra is very similar to the model in UPDM.

**Figure 5:**
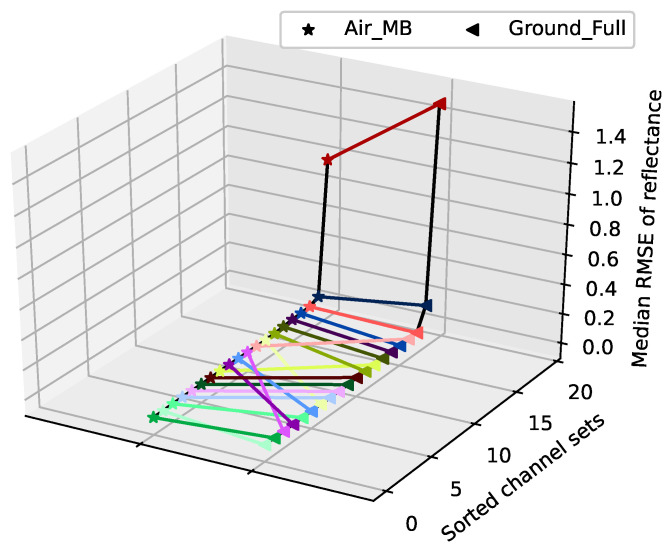
Applicability of band selection between datasets. Training was performed on dataset *Air_MA*.

In a last step before the main evaluation, we considered the role of band selection. Rather than developing a sophisticated algorithm and tuning its parameters as in [19], we concentrated on evaluating how well a band optimization on one dataset may be transferable to another. The result is shown in Figure 5: the dictionary was trained on *Air_MA* and evaluated for 20 random band sets on the datasets *Air_MB* and *Ground_Full*. The figure shows the resulting RMSE for all band sets sorted according to the RMSE. Each band set is denoted by a different color and the same band sets are connected with straight lines to visualize how the order of set quality correlates between datasets. Firstly, as found in [19], there are few very badly performing band sets. The remaining bands show similar performances. Now, taking the corresponding position of the sets between datasets into consideration, the high RMSE sets are rejected quite effectively by choosing some of the low RMSE filter sets. However, in the plateau, there are many non-parallel lines, indicating that choosing one of the low RMSE sets is likely to be far less optimal in the other dataset. Therefore, we refrain from using a more sophisticated algorithm and simply selected some of the good bands in the following evaluations, as this brings a major part of the improvements with far less effort.

Overall, we found that the training size has a relatively low influence on the result while the sparsity target and even the dictionary size should surprisingly be chosen to be low. More precise values will be further investigated in Section 5.

## 5. Results

In this section, we compare the performance of K-SVD against the other approaches in order to determine which is the best choice and under what circumstances. In contrast to the previous examples, here, training is always performed on a dataset using a different sensor than the dataset used for evaluation in order to better reflect the real-world situation. The selection of best bands is also performed on one of the sub-datasets using the same sensor as the training sub-dataset because the selection of best bands belongs to the training phase.

In each dataset combination, we compared the resulting RMSE for all pixels in the dataset with 20 replications for band selection and in the case of DCS, for the groups of pixels evaluated together. For K-SVD, we kept varying the dictionary size in order to further investigate which size is appropriate.

**Figure 6:**
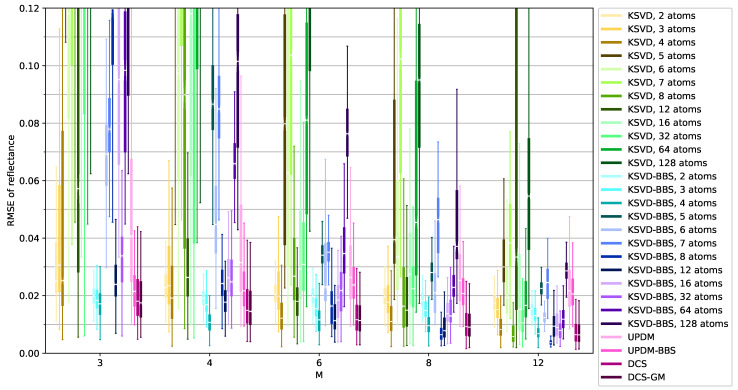
The evaluation results for all approaches on the training set *Air_MA*, band selection dataset *Air_MB* and evaluation dataset *Ground_Full*.

**Figure 7:**
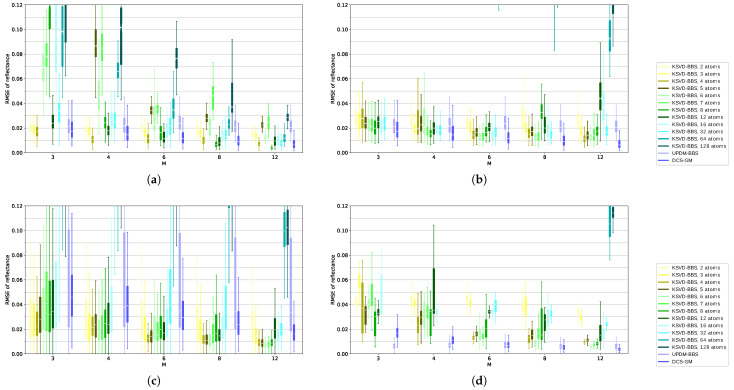
The evaluation results for different dataset combinations. (**a**) Training: *Air_MA*, Band Selection: *Air_MB*, Evaluation: *Ground_Full*, (**b**) Training: *Air_V1*, Band Selection: *Air_V2*, Evaluation: *Ground_Full*, (**c**) Training: *Ground_V1*, Band Selection: *Ground_V2*, Evaluation: *Air_Full*, (**d**) Training: *Ground_V1*, Band Selection: *Ground_V2*, Evaluation: *Air_V1*.

The results are shown in Figure 6 and Figure 7 for a varying number of bands *M* as box plots. Note that we refrained from including outliers in the graphics as they were highly distracting due to the sheer number as a result of the large sample size. For some parameters, the boxes partially or completely lie outside the plotting range. The first setting shown in Figure 6 is the main use case followed in the paper: training on a remote sensing image with a diverse environment and using it on measurements from the ground. We compared a total of six different approaches: *K-SVD*, *KSVD-BBS*, *UPDM*, *UPDM-BBS*, *DCS* and *DCS-GM*. *KSVD-BBS* refers to K-SVD including the best band selection; herein, the three best band sets according to the band selection sub-dataset were kept. *UPDM-BBS* refers to UPDM including the best band selection; again, the three best band sets were kept. *DCS-GM* refers to DCS with the mixing of the groups by calculating the median of all spectra calculated for one pixel. As the 20 replications are generated by combining five group selections with four band set selections, four median spectra of five spectra each were constructed per parameter set and pixel.

*K-SVD* and *KSVD-BBS* were performed with different numbers of atoms as shown in Figure 6. The main observations here are that an increasing number of bands naturally leads to a reduced RMSE for all approaches. The selection of the best bands in *KSVD-BBS* leads to a significant improvement in the *K-SVD* results. In addition to the lowered median, the spread of values is also much lower. The selection of the best bands in *UPDM-BBS* leads to a decent improvement, especially for small numbers of bands. The group mixing in *DCS-GM* also leads to a slight but reliable reduction in the RMSE in comparison to the pure *DCS* proving the benefit of this improvement. These improvements were very similar for all dataset combinations. Therefore, we refrained from including the un-improved versions in Figure 7 to make it more comprehensible and facilitate the comparison of the dataset combinations.

The first plot Figure 7a shown in Figure 7 is the same as in Figure 6 but only a more compact version—included here for facilitating the comparison. In this dataset combination, *DCS-GM* reliably delivers good results and outperforms *UPDM-BBS*. *KSVD-BBS* beats *DCS-GM* at certain dictionary sizes. Interestingly, for less than six bands, it performs best with approximately four atoms; while for more than six bands, it performs best with eight atoms. For all numbers of bands, *KSVD-BBS* is able to outperform *DCS-GM* for the best fitting number of atoms. However, even when selecting a slightly incorrect number of atoms, *DCS-GM* tends to be better.

**Figure 8:**
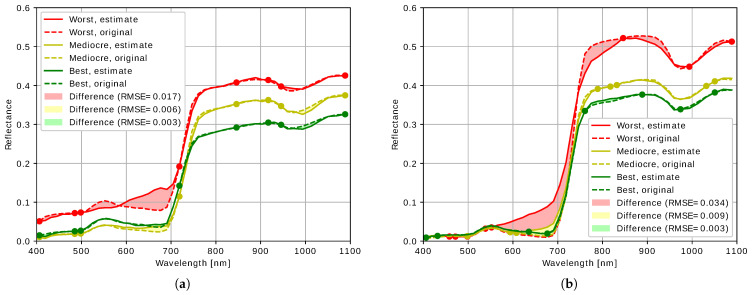
Selected reconstructed spectra from *Ground_Full*. (**a**) *KSVD-BBS*, (**b**) *DCS-GM*.

In order to provide a better understanding of the results, we picked the spectra with the lowest, highest and median RMSE from one of the simulations with eight bands and in the case of *KSVD-BBS*, eight atoms, for both *KSVD-BBS* and *DCS-GM*, are shown in Figure 8. For *KSVD-BBS*, the band set which performed best in the band selection dataset (*Air_MB*) was chosen. For *DCS-GM*, the band sets were randomly picked as no such indicator was available. In both approaches, for both the best case as well as the median, the differences of the estimate in comparison to the original spectra were very low, qualitatively confirming the quantitative findings.

This allows an estimate of the impact on vegetation indices: these are usually built by comparing the reflectance at different wavelengths. For a vegetation index which requires the reflectances at wavelengths which have not been directly measured, the reconstruction results clearly deliver better reflectance values for these wavelengths than simply using the closest measured values or an interpolation between the closest measured values. Hence, the result of the vegetation index will also be improved.

For the cases considered herein, the errors with *KSVD-BBS* and *DCS-GM* are qualitatively similar, not allowing the derivation of a general statement on the cause of the difference between the two approaches. However, the differences are much clearer in the worst-case spectrum. In both approaches, errors in the worst-case spectrum become the worst in the spectral ranges with few bands. In the case of *DCS-GM* (Figure 8b), these ranges are wider due to the many different band sets. One of the particularly uneven band distributions generates the worst case seen herein with five bands clustered in the range between 450 nm and 570 nm. In the case of *KSVD-BBS* (Figure 8a), the band selection is not as uneven because such band sets are rejected. While the relatively large gap with no bands between 500 nm between 700 nm causes the problems in the worst case, it cannot be identified as a fundamental problem because it only affects the worst cases. In the median and best case, this gap has little to no impact on the reconstruction quality. Note that the three representatives in both Figure 8a,b were selected from the same set of pixels. Therefore, the reflectance being overall higher in Figure 8b is completely coincidental.

In Figure 7b,d, we investigated the performance when training and evaluating on datasets containing solely vegetation data. In Figure 7b, training was performed on the remote sensing dataset and evaluation was performed on the ground dataset. In Figure 7d, it was the other way around. In both cases, *DCS-GM* is ahead as it performs particularly well with homogeneous data. Interestingly, in Figure 7b, *KSVD-BBS* is superior over *UPDM-BBS* despite suffering from the same restriction with both the training for *KSVD-BBS* and the base spectra for *UPDM-BBS* being based on the other dataset. In Figure 7d, *UPDM-BBS* performs almost as good as *DCS-GM* for high band numbers and superior for small band numbers, as found in [2], which may be explained by very similar vegetation spectra in the dataset *Air_V1* that are also very similar to the base spectra in use. In Figure 7d, *KSVD-BBS* performs slightly better than in Figure 7b at least for a higher number of bands and optimal number of atoms. Together with Figure 7a, this shows a trend of *KSVD-BBS* benefiting from more diversified training data. However, in Figure 7d, *DCS-GM* and *UPDM-BBS* benefit more from the similar data, rendering *KSVD-BBS* inferior in this case. The similarity also leads to the very low variation of the results with all approaches.

Figure 7c was included mainly for the completion of the dataset combinations. Its sense is limited as the learning dataset is far less diverse than the evaluation dataset. However, *KSVD-BBS* still works surprisingly well in this case in comparison to *DCS-GM* and *UPDM-BBS* which both fall behind in this case because of the lower similarity between pixels in the case of *DCS-GM* and less vegetation pixels in the case of *UPDM-BBS*. Since *Air_Full* is a typical remote sensing dataset, this shows that *KSVD-BBS* may also be a promising approach for estimating hyperspectral remote sensing images based on multispectral remote sensing images—aside from its application in M2HSNs.

**Figure 9:**
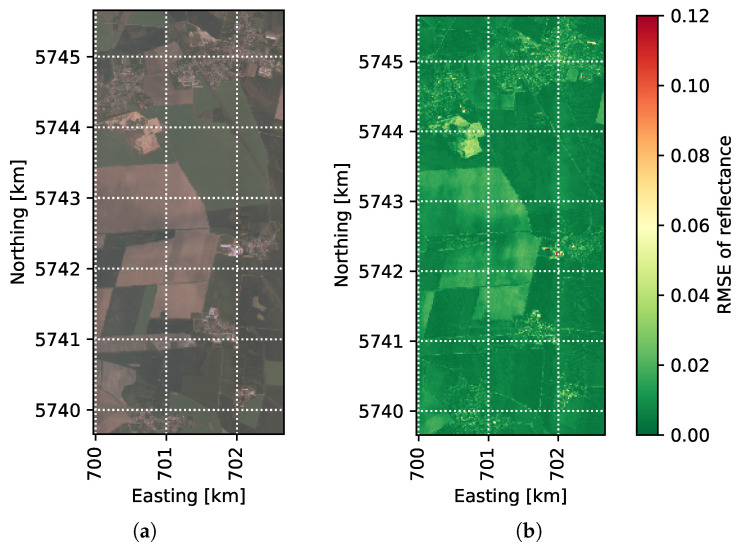

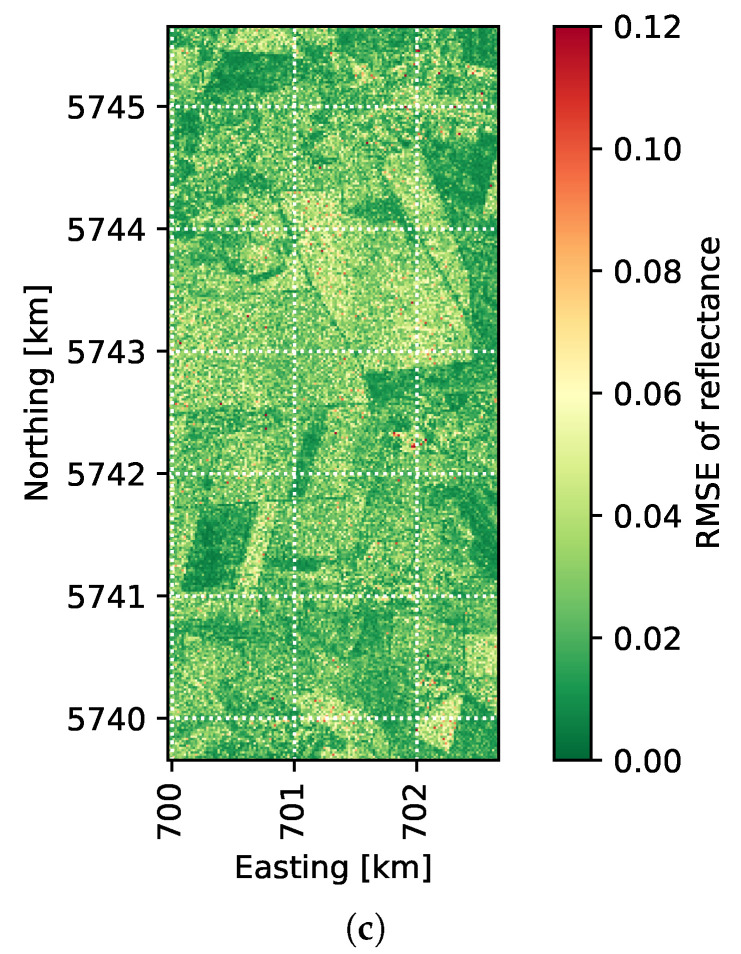
Investigation of the reconstruction quality on the area of *Air_Full*. The coordinates are in UTM zone 32U. (**a**) Map with normal coloring (see [2]). (**b**) Resulting RMSE with *KSVD-BBS*. (**c**) Resulting RMSE with *DCS-GM*.

For a qualitative evaluation of this case, we assess the reconstruction quality across the area in Figure 9. Figure 9a is an RGB image including the corresponding bands from the original dataset. Figure 9b,c show the RMSE per pixel for one of the best band sets according to training in *KSVD-BBS* and a group mixing randomly selected for *DCS-GM*. In Figure 9b, *KSVD-BBS* generates low RMSE values especially in vegetation areas. Curiously, the trained base was also suitable for the bare soil areas, although no such samples were included in the training base. The quality suffers in the villages. If planning to use the approach on remote sensing data, clearly a more diversified training dataset is required. Figure 9c shows why the RMSE values are higher with *DCS-GM*: the quality is only about as good as with *KSVD-BBS* in some of the vegetation areas but the main effect affecting the RMSE is the extreme variation across the whole area. We attribute this to a mixture of two effects: the first one is the one observed in Figure 8b, that in some pixels an uneven bands selection leads to bad reconstructions. The second effect is that the groupings often include spectra of differing kinds which reduce the overall reconstruction quality of the group. The latter effect also serves as the main explanation for why the reconstruction quality is significantly better in all the other scenarios with less variation across the spectra.

## 6. Discussion and Future Work

In this paper, we found that K-SVD can indeed be used for obtaining hyperspectral data from multispectral data for vegetation data obtained via remote sensing or in situ. In comparison to DCS, it does not require using different sensors and instead relies on a learned dictionary which can be kept remarkably simple. For very similar data, DCS and UPDM deliver superior results. On data with a certain degree of variation, which is more likely to be the target of investigations, K-SVD delivers similar or even better results than DCS. However, this requires the more careful tuning of parameters, namely the bands used and the dictionary size. It even works if being trained with datasets from different sensors, which makes the acquisition of training data relatively easy. Furthermore, K-SVD showed potential for estimating hyperspectral remote sensing images based on multispectral remote sensing images. In our future work, we were planning to evaluate the M2HSN approach using DCS and K-SVD in a real-world deployment and to investigate the application of K-SVD on remote sensing data more closely. We are also planning to quantitatively investigate the impact of the reconstruction quality on data derived from the spectra.
